# Aneurysm severity is suppressed by deletion of CCN4

**DOI:** 10.1007/s12079-021-00623-5

**Published:** 2021-06-02

**Authors:** Helen Williams, Kerry S. Wadey, Aleksandra Frankow, Hazel C. Blythe, Tessa Forbes, Jason L. Johnson, Sarah J. George

**Affiliations:** Translational Health Sciences, Bristol Medical School, Research Floor Level 7, Bristol Royal Infirmary, Bristol, BS2 8HW UK

**Keywords:** CCN-4, WISP-1, WISP1, Aneurysm, Atherosclerosis, Wnt pathway

## Abstract

Patients with abdominal aortic aneurysms are frequently treated with high-risk surgery. A pharmaceutical treatment to reverse aneurysm progression could prevent the need for surgery and save both lives and healthcare resources. Since CCN4 regulates cell migration, proliferation and apoptosis, processes involved in aneurysm progression, it is a potential regulator of aneurysm progression. We investigated the role of CCN4 in a mouse aneurysm model, using apolipoprotein-E knockout (ApoE^−/−^) mice fed high fat diet and infused with Angiotensin II (AngII). Blood pressure was similarly elevated in CCN4^−/−^ApoE^−/−^ mice and CCN4^+/+^ApoE^−/−^ mice (controls) in response to AngII infusion. Deletion of CCN4 significantly reduced the number of ruptured aortae, both thoracic and abdominal aortic area, and aneurysm grade score, compared to controls. Additionally, the frequency of vessel wall remodelling and the number of elastic lamina breaks was significantly suppressed in CCN4^−/−^ApoE^−/−^ mice compared to controls. Immunohistochemistry revealed a significantly lower proportion of macrophages, while the proportion of smooth muscle cells was not affected by the deletion of CCN4. There was also a reduction in both proliferation and apoptosis in CCN4^−/−^ApoE^−/−^ mice compared to controls. In vitro studies showed that CCN4 significantly increased monocyte adhesion beyond that seen with TNFα and stimulated macrophage migration by more than threefold. In summary, absence of CCN4 reduced aneurysm severity and improved aortic integrity, which may be the result of reduced macrophage infiltration and cell apoptosis. Inhibition of CCN4 could offer a potential therapeutic approach for the treatment of aneurysms.

**Supplementary Information:**

The online version contains supplementary material available at 10.1007/s12079-021-00623-5.

## Introduction

Abdominal aortic aneurysms occur when the wall of the aorta loses integrity, leading to the aorta bulging from a normal diameter of 2 cm, to more than 3 cm. Currently aneurysms are surgically treated when their diameter exceeds 5.5 cm, as they are considered to have a high risk of rupture (Aggarwal et al. 2011). NICE guidelines (2008) state that 20% of 5 cm vessels and 25% of 6 cm vessels will rupture every year. However the surgery is also high risk and results in 5% mortality for open surgery and 1–2% for endovascular repair (Kim et al. 2016). A pharmaceutical therapy to either reverse or retard aneurysm progression could prevent aneurysms from rupturing as well as the need for surgery, saving both lives and healthcare resources.

CCN4 is a secreted signalling protein member of the CCN family (Perbal and Perbal 2016) and is a Wnt signalling pathway target gene and therefore is also sometimes referred to as WISP-1. It has various effects on cell behaviour, including being pro-adhesion, migration, proliferation, differentiation and survival (Jun and Lau 2011; Liu et al. 2013a, b). CCNs also have roles in pathologies such as fibrosis, inflammation and cancer (Kim et al. 2018). The progression of aneurysms is the result of cell migration, proliferation and apoptosis. Indeed CCN3 has been shown to protect against aneurysms (Zhang et al. 2016), with overexpression viruses lessening the severity of disease, while knockout of CCN3 increased aneurysm severity.

CCN4 has many roles in the cardiovascular system and in cells that are involved in aneurysm formation, including vascular smooth muscle cells (VSMCs) and macrophages. Liu et al. (2013a, b) showed that in cultured rat aortic VSMCs, CCN4 was induced by TNFα and that increasing CCN4 using a recombinant protein led to increased cell migration and proliferation; this migratory effect was mediated via integrin α5β1. Williams et al*.* (2016) demonstrated that CCN4 was pro-migratory in mouse VSMCs, following its β-catenin-dependent induction by Wnt2, both in vitro and in vivo, using the mouse carotid artery ligation model.

Marchand et al. (2011) and our own group (Mill et al. 2014; Williams et al. 2016) confirmed the presence of CCN4 in both human arterial cells and atherosclerotic lesions, while Reddy et al. (2011) showed that IL-18 increased survival and proliferation via TCF/LEF and CCN4 in human VSMCs. However, CCN4 then acted via AP-1 to upregulate MMPs 2, 9 and 14. This could, conversely, lead to increased proteolysis and breakdown of tissues and vulnerability to aneurysm. This increase in tissue turnover rate is likely involved in tissue repair, but the effect of changes to this on risk of aneurysm is as yet unknown. There is also conflicting data regarding whether CCN4 is likely to be higher or lower in those at risk of aneurysm. CCN4 positively correlated with both weight, dietary fat (Murahovschi et al. 2015) and leptin (Tacke et al. 2018) in patients, suggesting it would be increased in those vulnerable to aneurysm. While conversely the CREB-dependent upregulation of CCN4 was lost with age in mice, so as aneurysm susceptibility increases with age, these patients may have lower active levels (Brown et al. 2019).

Evidence is also emerging that CCN4 promotes monocyte recruitment, as well as increasing the inflammatory response in macrophages following injury and to promote wound repair. Elevated CCN4 induced a pro-inflammatory response in macrophages, inducing production of cytokines, increasing levels of MMPs and inducing a more inflammatory phenotype (Murahovschi et al. 2015). Similarly in a mouse model of osteoarthritis, stimulation of macrophages with CCN4 resulted in production of MMPs 3, 9 and 13 (Blom et al. 2009); while in mucosal epithelial cells macrophages released IL-10, which increased CCN4 via CREB activation and resulted in wound repair. In fibroblasts CCN4 induced VCAM-1 expression via the Syk, PKCδ, JNK, c-Jun, and AP-1 signalling pathways; this increased VCAM-1 expression, then promoted monocyte adhesion to the fibroblasts (Liu et al. 2013a, b). The actions of CCN4 in cell types ranging from cancer cell lines to fibroblasts are mediated by a variety of integrins, including αVβ5, αVβ3 and β1 (Chuang et al. 2013; Liu et al. 2013a, b; Ono et al. 2011; Stephens et al. 2015).

These multiple potential roles for CCN4 in the processes associated with aneurysm progression indicate that CCN4 deletion may suppress aneurysm formation. To directly investigate the role of CCN4 in aneurysm formation Apoliprotein E deficient (ApoE^−/−^) mice were crossed with CCN4 null mice to generate ApoE^−/−^CCN4^−/−^ mice and CCN4 wildtype ApoE deficient controls (ApoE^−/−^CCN4^+/+^). Mice received Angiotensin II infusion and high fat diet feeding to induce aneurysm formation and the effect of CCN4 deletion was evaluated. These in vivo studies were supplemented with in vitro analysis of the effect of CCN4 on monocyte/macrophage adhesion and migration.

## Methods

### Animals

The housing and care of all the animals and the procedures used in these studies were performed in accordance with the guidelines and regulations of the University of Bristol and the United Kingdom Home Office. The investigation conforms to the *Guide for the Care and Use of Laboratory Animals* published by the US National Institutes of Health (NIH Publication No. 85-23, revised 1996).

#### Transgenic mice

CCN4 homozygous knockout mice (CCN4^−/−^) backcrossed onto C57bl/6J for 10 backcrosses, were a kind gift from Marian Young (NIH, Bethesda, MD). ApoE^−/−^ mice were purchased from Charles River UK (criver.com). Mice were bred at the University of Bristol breeding facility to produce CCN4^−/−^ApoE^−/−^ double knockout mice and CCN4^+/+^ApoE^−/−^ single knockout littermate controls. All mice used in the study were ApoE^−/−^, but the labels CCN4^−/−^ and CCN4^+/+^ are used in figures for simplicity, with full nomenclature in figure legends.

#### Induction of aneurysms

Male 8 week old CCN4^−/−^ApoE^−/−^ and CCN4^+/+^ApoE^−/−^ mice were fed a high fat diet containing 21% (w/w) fat and 0.15% (w/w) cholesterol (IPS custom diet, testdiet.com). Following 4 weeks of high fat diet, blood pressure was measured (Kent scientific CODA blood pressure measurement system, kentscientific.com) and subsequently animals were anaesthetised with inhaled 3% isofluorane in oxygen and an osmotic minipump (Alzet model 2004, alzet.com) was inserted subcutaneously on the back of the neck using the model created by Daugherty et al. (2000). Pumps dispensed 500 ng/kg/min AngII (Enzo life sciences, enzolifesciences.com) for 28 days. Post-operative analgesia was achieved by an IP injection of 0.1 mg/kg buprenorphine. After 28 days, blood pressure was measured prior to termination. Animals were anaesthetised by IP injection of sodium pentobarbitone (500 mg/kg) and under terminal anaesthesia the animals were perfuse fixed by constant pressure perfusion at 100 mmHg using PBS followed by 10% formalin in PBS. Outflow of fixative was via transected jugular veins.

### Histochemistry, immunohistochemistry and immunofluorescence

Aortae were removed following perfusion fixation, further fixed in 10% formalin in PBS for 24 h and photographed. They were then cut into 4 thoracic and 4 abdominal sections and embedded in agar prior to processing and embedding in paraffin wax. 3 μm sections were cut and mounted upon Superfrost slides for elastin van Gieson (EVG) staining and upon Superfrost Plus slides for immunohistochemistry and immunofluorescence. EVG stained sections were assessed by image analysis (Image Pro). The mean aneurysm grade score was calculated from the aortic segment exhibiting the highest degree of aneurysm (most dilated/diseased section per aorta graded). The presence of vessel wall remodelling on the aorta (fibrous adventitial thickening) in any of the eight segments of the aortae was identified. The number of elastin breaks was quantified from the average of 4 thoracic or 4 abdominal aortic segments. Areas of lumen, media, adventitia, IEL and EEL were measured in all eight segments and the average thoracic and abdominal aortic sections calculated.

Immunohistochemistry was performed to visualise α-smooth muscle actin (Sigma, A2547, 3.1 μg/ml using the Vector MOM kit), CCN4 (R&D AF1680 1 μg/ml), desmin (R&D AF3844 2 μg/ml) apoptosis (cleaved PARP, Abcam, ab32064, 4.8 μg/ml), cleaved caspase 3 (R&D AF835 1 μg/ml) and proliferation (PCNA, Abcam, 18197, 1 μg/ml). Non-immune IgG of the same species as the primary was used as negative control in all protocols at the same concentration as the primary antibody to demonstrate the specificity of the protocol. Macrophages were detected by GSL lectin staining (Vector Labs, B1205, 2.5 μg/ml). For dual staining sections were first immunostained for apoptosis (Cleaved PARP, Abcam, ab32064, 4.8 μg/ml), proliferation (PCNA, Abcam, 18197, 1 μg/ml) or CCN4 (R&D, AF1680 1 ug/ml) before double staining for VSMCs (α-smooth muscle actin, Sigma, A2547, 3.1 μg/ml) sing the Vector MOM kit (Vector Laboratories BMK-2202).

### In vitro study of macrophages

#### Isolation of human monocytes

Peripheral blood was collected from healthy human volunteers in accordance with the Regional Ethics Committee (NRES #10/H0107/32). Blood was diluted with an equal volume of sterile PBS and stratified on Ficoll-Paque Plus (GE Healthcare 17-1440-02).

#### Adhesion of monocytes

Human umbilical cord endothelial cells (HUVECs) were grown to confluence and treated with 10 ng/ml TNF-α in the presence or absence of 2.5 μg/ml CNN4 for 24 h to activate the endothelial cells and enable monocyte adhesion. THP-1 cells (a monocyte cell line) labelled with 10 μM calcein AM (Sigma 17703) were applied to the treated HUVECs for 30 min before washing to remove non-adherent cells. Adherent cells were photographed and quantified.

#### Western blotting and ELISA

HUVECs treated with 10 ng/ml TNF-α and ± 2.5 μg/ml CCN4 for 24 h were lysed using 5% SDS lysis buffer and equal amounts of protein were loaded onto a gel. After transfer onto a nitrocellulose membrane, 0.061 mg/ml ICAM-1 (Abcam ab53013), 0.437 mg/ml VCAM-1 (Abcam ab134047), 0.5 mg/ml E-selectin (Abcam ab18981) and 0.0635 mg/ml P-selectin (Abcam ab182135) antibodies were added overnight at 4 °C, followed by incubation with secondary antibodies for 1 h at RT and detection was achieved using ECL and a Bio-Rad densitometer. Conditioned media was collected after 24 h and interleukin-6 (IL-6) protein quantified by ELISA (R&D Systems), using the manufacturer’s instructions.*Macrophage migration.*

The peripheral blood mononuclear cells were collected, washed and seeded in 20 ng/ml M-CSF in 10% FCS/RPMI 1640. The resultant adherent monocyte cells were matured by culture for 7 days before use. Macrophages were seeded in a 24-well plate transwell (Millipore, 141006) in the presence or absence of 0.5 μg/ml recombinant CCN4 and with 20 ng/ml MCP-1 in the bottom of the tissue culture plate well. After culture for 48 h migrated macrophages were stained with haematoxylin and quantified.

### Statistics

Results are expressed as mean + SEM. All data was checked for normal distribution and were analysed by Student’s *t* test when normally distributed, or Mann–Whitney test when non-normally distributed. For multiple comparisons ANOVA with Student Newman Keuls post-test and for contingency testing Chi squared test was used. Significant difference was accepted when *p* < 0.05.

## Results

### Blood pressure was increased by AngII

**Fig. 1:**
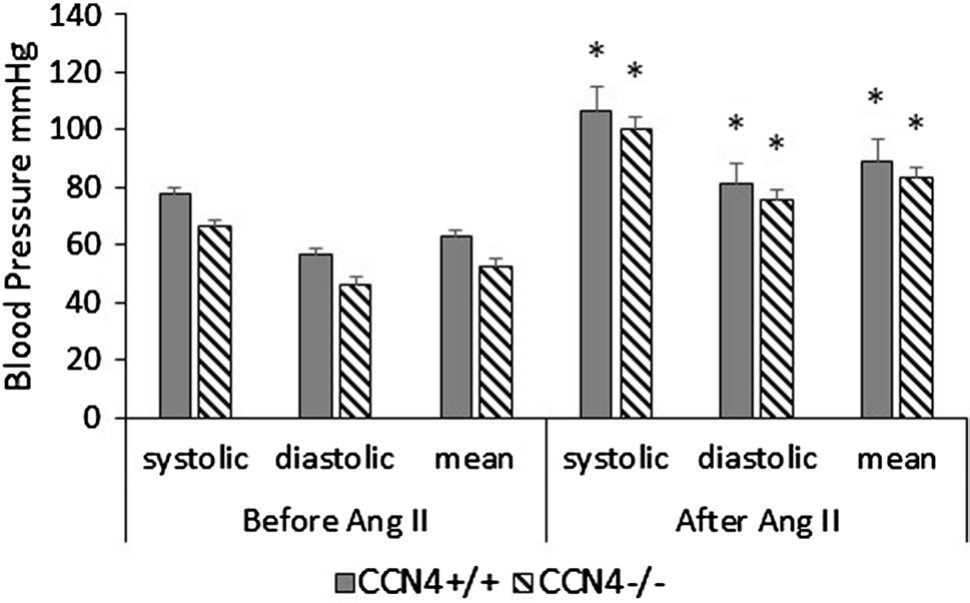
AngII increased blood pressure in CCN4^+/+^ApoE^−/−^ and the CCN4^−/−^ApoE^−/−^ mice. CCN4^−/−^ApoE^−/−^ and wild type CCN4^+/+^ApoE^−/−^ mice were infused with Angiotensin II (AngII) for 28 days using mini-osmotic pumps. Blood pressure was measured on day 0 (before AngII) and day 28 (after AngII). Data is presented as mean ± sem, CCN4^−/−^ApoE^−/−^ n = 13 and CCN4^+/+^ApoE^−/−^ n = 14, * indicates p < 0.001 compared to before AngII controls, ANOVA

As we expected infusion of AngII significantly increased systolic, diastolic and mean blood pressure in both the CCN4^−/−^ApoE^−/−^ knockout mice and CCN4^+/+^ApoE^−/−^ controls (Fig. 1). Importantly, there was no significant difference in either the systolic, diastolic or mean blood pressure in the CCN4^−/−^ApoE^−/−^ knockout mice and CCN4^+/+^ApoE^−/−^ controls either before or after AngII infusion (ANOVA with Student Newman Keuls post-test).

### Knockout of CCN4 reduced aneurysm severity

**Fig. 2:**
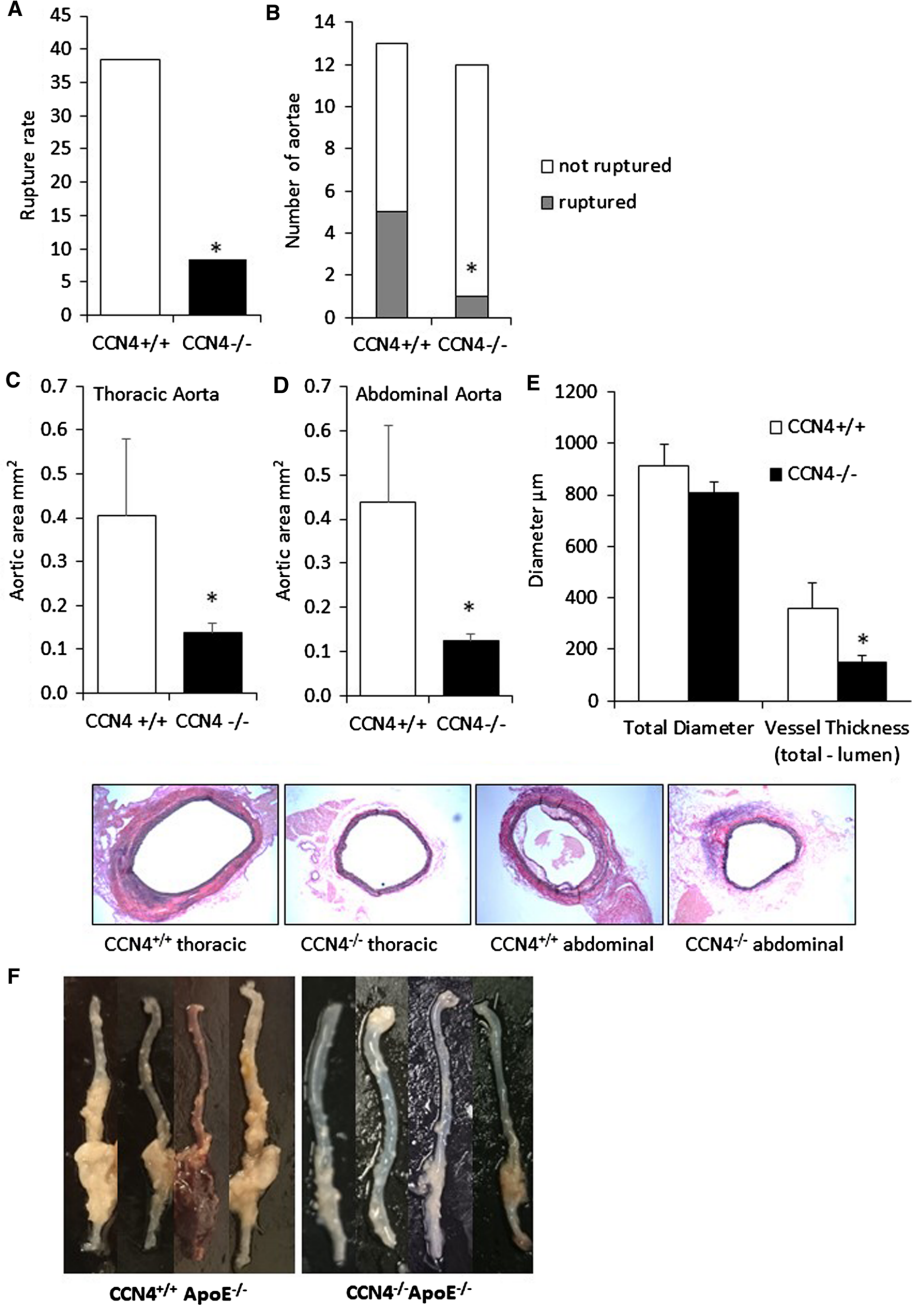
CCN4 deletion reduced rupture incidence, aortic size, and vessel thickness. Thoracic and abdominal aortae sections from CCN4^−/−^ApoE^−/−^ and CCN4^+/+^ApoE^−/−^ mice exposed to AngII for 28 days were stained with EVG and aortic area measured by image analysis. CCN4 deletion reduced the rupture rate (**a**), number of ruptured aortae (**b**), average thoracic (**c**) and average abdominal (**d**) aortic size and average vessel thickness (**e**). Representative images of transverse sections through thoracic and abdominal aortae and images of gross anatomy (**f**) are included. Data is presented as mean ± sem, CCN4^+/+^ApoE^−/−^ n = 13 and CCN4^−/−^ApoE^−/−^ n = 12, * indicates *p* < 0.05 compared to CCN4+/+ controls, all Mann–Whitney test except (B) Chi squared test

**Fig. 3:**
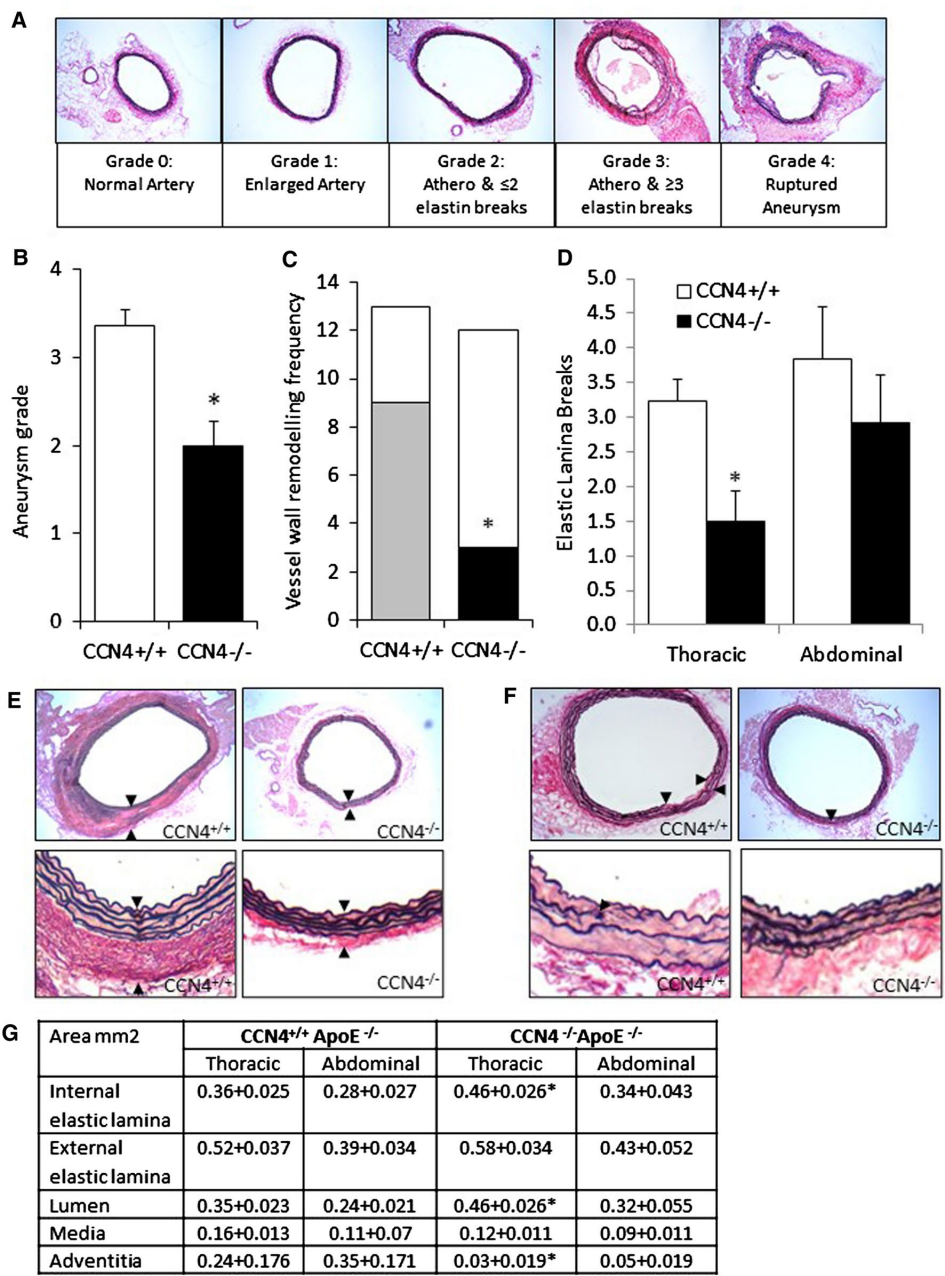
CCN4 deletion reduced AAA formation. Aneurysm grade score—representative images of aortic sections stained with EVG and identified as grade 0–4. ApoE^−/−^CCN4^−/−^ and ApoE^−/−^CCN4^+/+^ mice were exposed to AngII for 28 days and aortic sections stained with EVG (**a)**. The mean aneurysm grade score was calculated from the aortic segment exhibiting the highest degree of aneurysm (most dilated/diseased section per aorta graded) (**b)**. The presence (grey/black bars) and absence (white bars) of vessel wall remodelling on the aorta (presence of fibrous adventitial thickening, as seen in grade 3 example, in any of the sections along the aorta) was noted (**c**). The number of elastin breaks was quantified from the average of 4 thoracic or 4 abdominal aortic segments (**d**). Representative images of EVG stained aortae to illustrate vessel wall remodelling (**e)** and elastin breaks (**f)**, indicated by arrowheads at both low and high power. **g** Physical parameters of the average thoracic and abdominal aortic sections. CCN4^+/+^ApoE^−/−^ n = 13 and CCN4^−/−^ApoE^−/−^ n = 12, * indicates *p* < 0.05 compared to CCN4 + / + controls. Mann–Whitney test for aneurysm score, elastin breaks and physical parameters, data is presented as mean ± sem (B and D); Fisher’s Exact test for adventitial thickening (C)

The rupture rate, number of ruptured aneurysms, vessel thickness and vessel wall area in both the thoracic and abdominal portions of aortae were all significantly reduced in the CCN4^−/−^ApoE^−/−^ mice compared to CCN4^+/+^ApoE^−/−^ controls (Fig. 2a–f). This indicates that aneurysm progression was retarded in the aortae of CCN4^−/−^ApoE^−/−^ mice compared to control mice. This is confirmed by the significant reduction in the average aneurysm grade score (using the scoring system illustrated in Fig. 3a and measuring the largest aortic section) in CCN4^−/−^ApoE^−/−^ compared to control mice (Fig. 3b). Additionally, the number of aortae exhibiting vessel wall remodelling, clearly identified as a fibrous thickening exterior to the media, and the presence of elastic laminae breaks (measured in the largest thoracic and abdominal section) were assessed as indicators of aneurysm progression (Fig. 3c, d). We chose these as measures of aneurysm progression as in this model they are the most striking features when looking at the sections and appear to be important stages of aneurysm progression. Deletion of CCN4 was associated with a significant decrease in these parameters compared to control (Fig. 3c, d). There were no significant differences in the size of the intima, media or adventitia in the aortae collected from CCN4^−/−^ApoE^−/−^ and CCN4^+/+^ApoE^+/+^ mice prior to treatment with AngII (Supplemental Fig. 1).

### Knockout of CCN4 reduced macrophage but not VSMC content in vivo

**Fig. 4:**
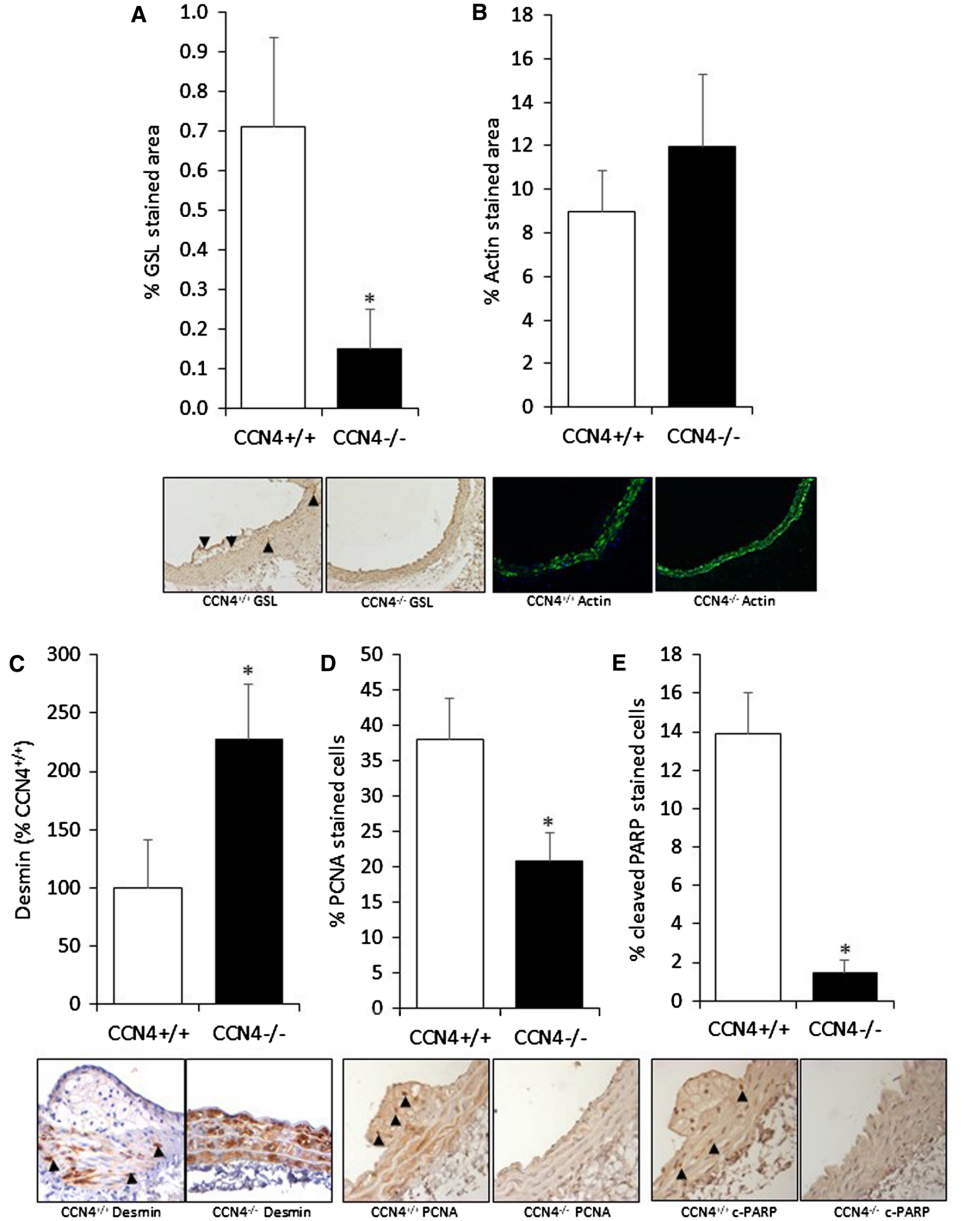
Effect of CCN4 deletion on macrophage and VSMC content, desmin, proliferation and apoptosis markers. ApoE^−/−^CCN4^−/−^ and ApoE^−/−^CCN4^+/+^ mice were exposed to AngII for 28 days. Macrophage content was quantified by GSL staining (**a**) and VSMC content was quantified by α-smooth muscle actin immunofluorescence (**b**) in aortic sections. Desmin protein was quantified and as a percentage of the amount of desmin in CCN4^+/+^ mice (**c**). Proliferation was quantified by PCNA immunohistochemistry (**d**) and apoptosis was quantified by cleaved PARP immunohistochemistry (**e**) in aortae sections. * indicates p < 0.05 compared to CCN4^+/+^ controls, Mann–Whitney test. CCN4^+/+^ApoE^−/−^ n = 13 and CCN4^−/−^ApoE^−/−^ n = 12. Positive cells are brown and indicated with arrowheads, except for actin where positive cells are green, in the shown representative images

Analysis of the composition of the aortae demonstrated a significant reduction in the macrophage content of aortae from CCN4^−/−^ApoE^−/−^ mice compared to CCN4^+/+^ApoE^−/−^ controls (Fig. 4a). However, it is important to note that macrophage content is very low in both cases, accounting for less than 1% of the vessel. There was no significant difference in the VSMC content (Fig. 4b).

### Knockout of CCN4 increased desmin content in the aortae

Desmin was significantly increased in CCN4^−/−^ApoE^−/−^ mouse aortae compared to CCN4^+/+^ApoE^−/−^ controls (Fig. 4c), this indicates that the deficiency of CCN4 promotes the contractile VSMC phenotype.

### Knockout of CCN4 reduced proliferation and apoptosis in vivo

A significant reduction in the proportion of proliferating cells was detected in the aortae from CCN4^−/−^ApoE^−/−^ mice compared to CCN4^+/+^ApoE^−/−^ controls (Fig. 4d). Additionally, a significant reduction in the number of apoptotic cells detected using cleaved PARP (and confirmed using cleaved caspase 3 in supplemental Fig. 1c) was observed in the aortae from CCN4^−/−^ApoE^−/−^ mice compared to CCN4^+/+^ApoE^−/−^ controls (Fig. 4e). Dual immunostaining (Supplemental Fig. 2) showed that VSMCs were both proliferating and undergoing apoptosis in aortae from CCN4^+/+^ApoE^−/−^ controls.

Immunofluorescence for CCN4 protein showed that CCN4 was detected at low levels in the aortae from CCN4^+/+^ApoE^−/−^ mice in the absence of AngII (Supplemental Fig. 3). CCN4 protein levels were higher in CCN4^+/+^ApoE^−/−^ mice infused with AngII, with CCN4 protein detectable in the medial and adventitial layers. Since CCN4 is a soluble protein capable of diffusing through the aortic wall, medial VSMCs could be affected by medial and adventitial derived CCN4. As expected CCN4 protein was absent in aortae from CCN4^−/−^ApoE^−/−^ mice treated with AngII.

### Recombinant CCN4 enhanced monocyte adhesion and macrophage migration in vitro

**Fig. 5:**
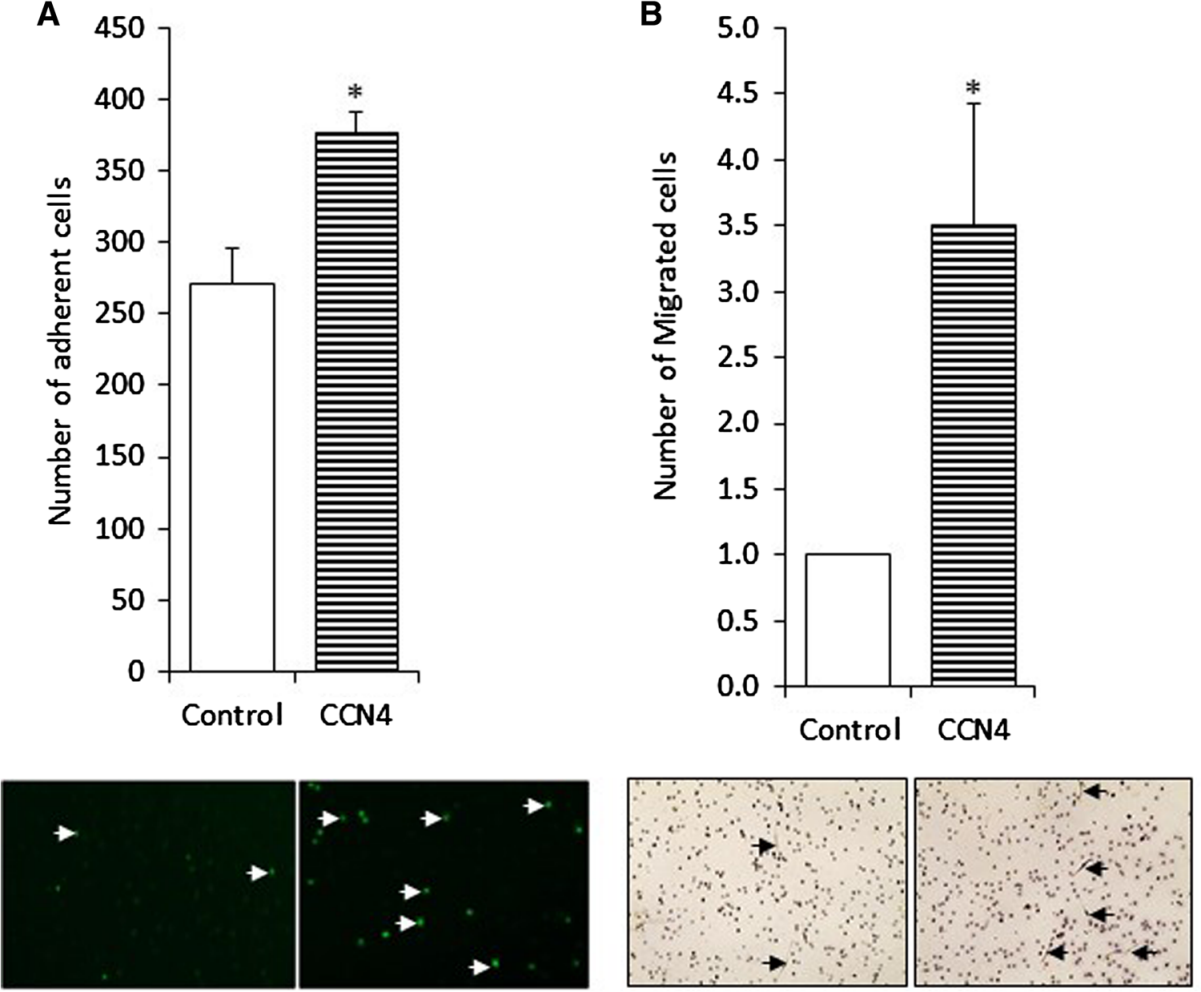
Induction of monocyte adhesion and macrophage migration in vitro by recombinant CCN4 protein. **a** Monocyte adhesion to endothelial cells was quantified following treatment of HUVECs with recombinant CCN4 protein. Representative images are shown beneath. * indicates *p* < 0.05 compared to control, Student’s t-test, n = 3. **b** Monocyte migration was quantified in the presence and absence of recombinant CCN4 protein. Representative images are shown beneath. * indicates *p* < 0.05 compared to control, one sample *t* test, n = 4

Recombinant human CCN4 significantly increased the number of monocytes adhering to human endothelial cells (Fig. 5a) and enhanced monocyte-derived macrophage migration (Fig. 5b). There was no significant difference in the amount of ICAM-1 and VCAM-1 proteins (supplemental Fig. 4, n = 6), or E-selectin and P-selectin proteins in endothelial cells cultured in the presence of CCN4 compared to control endothelial cells (data not shown, n = 3). However, CCN4 protein significantly enhanced IL-6 protein in the conditioned media collected from endothelial cells cultured with CCN4 compared to control endothelial cells (930.3 ± 11.7 vs.500.5 ± 4.6 pg/ml).

## Discussion

This study illustrates that deletion of CCN4 leads to a retardation of aneurysm progression in the thoracic and abdominal aortae of ApoE^−/−^ knockout mice. This was observed as a significant decrease in the number of ruptures, aortic vessel wall size and aneurysm grade, as well as a reduced frequency of other features of aneurysm progression (vessel wall remodelling and breaks in elastic lamellae). The suppression of features of aneurysm progression was associated with reduced numbers of macrophages and less proliferating cells within the aortic wall. Additionally, the number of apoptotic cells within the aortic wall was also reduced, which we propose may be due to a reduction in macrophage infiltration and activity; and a reduction in VSMC turnover. It is important to note that we had low levels of macrophages in both our experimental groups, so it is likely that the large changes in proliferation and apoptosis reflect changes in VSMC phenotype and rates of proliferation and apoptosis, this was confirmed by dual immunofluorescence for cleaved-PARP and the VSMC marker, a-smooth muscle actin. Vessel wall remodelling was observed in control mice in response to Ang II infusion and this was associated with enhanced presence of CCN4 in the aortae. We therefore suggest that the absence of CCN4 protein in CCN4 deficient mice retards vessel remodelling and is associated with the retention of the contractile VSMC phenotype, and reduced proliferation and apoptosis.

In vitro we observed that recombinant CCN4 promoted adhesion of monocytes to endothelial cells migration of macrophages, however the expression of adhesion molecules on endothelial cells (ICAM-1, VCAM-1, E-selectin and P-selectin) was not altered. In contrast CCN4 enhances IL-6 protein levels as previously observed by Hou and colleagues in fibroblasts (Hou et al. 2013), suggesting this contributes to the increased adherence and entry of monocytes into the aortic wall. In support of the pro-inflammatory role of CCN4, the absence of CCN4 in our in vivo model resulted in reduced macrophage content. We propose that the reduced presence of macrophages within the aortae results in a reduced apoptotic stimulus and therefore suppression of aneurysm progression that was observed. Our observation that CCN4 promotes macrophage migration is similar to the effect of CCN4 in VSMCs (Liu et al. 2013a, b; Williams et al. 2016). In support of our conclusion that CCN4 promotes monocyte adhesion to endothelial cells, Liu et al. (2013a, b) showed that CCN4 promoted monocyte adhesion to fibroblasts. (Hou et al. 2013b).

Elevated circulating levels of CCN4 could therefore be a contributing factor in the initiation of aortic aneurysms, particularly as both weight and BMI are risk factors for development of AAA and weight was correlated to CCN4 (Murahovschi et al. 2015). Accordingly, Tacke et al. (2018) demonstrated an inflammatory macrophage phenotype with increased CCN4, corroborating our data showing enhancement of rates of macrophage adhesion and migration by CCN4 in vitro and the higher proportion of macrophages observed within aortae of CCN4^+/+^ApoE^−/−^ mice compared to CCN4^−/−^ApoE^−/−^ mice in vivo.

CCN4 is known to increase the activity of various MMPs (Murahovschi et al. 2015; Reddy et al. 2011), whose proteolytic activity increased the propensity of aneurysm formation (Di Gregoli et al. 2017; Amin et al. 2016). It is therefore possible that the pro-aneurysmal effect of CCN4 occurs via upregulation of MMPs, which cause matrix degradation, and therefore contribute to the breakdown of elastic lamellae as well as cell apoptosis observed in our study.

It is known from the work of both our own group (Williams et al. 2016; Mill et al. 2014) and others (Ono et al. 2011, 2018; Chuang et al. 2013; Hou et al. 2013a; Liu et al. 2013a, b; Liu et al. 2013a, b) that CCN4 mediates it’s effects at least in part by integrins. Stephens et al. showed that αVβ5, αVβ3 and β1 integrins were involved in binding of CCN4 to A549 cells. Additionally, α5β1 commonly mediates CCN4 effects of downstream actions such as migration and proliferation (Liu et al. 2013a, b; Ono et al. 2011), however in some studies αVβ1 and α6β1 have also been suggested for inflammatory roles, such as IL-6 production (Hou et al. 2013a) and monocyte adhesion via an increase in VCAM-1 (Liu et al. 2013a, b).

It is not surprising that deletion of CCN4 results in the opposite effect to that seen when deleting CCN3 (Zhang et al 2016), as although CCN3 and 4 are from the same family of molecules, they have opposing effects both in vitro and in vivo. CCN3 reduced endothelial inflammation (Lin et al. 2010) and reduced VSMC proliferation and migration (Abe et al. 2010; Shimoyama et al. 2010) and neointimal formation in vivo (Shimoyama et al. 2010) whilst opposing effects are observed with CCN4.

In conclusion, we demonstrate that CCN4 is a contributing factor to development of AAA. If a topical or pharmaceutical therapy could be developed to suppress expression or inhibit CCN4, progression of AAA could be attenuated and thus reduce the need for high risk and invasive surgery.

## Supplementary Information

Below is the link to the electronic supplementary material.

(A) Representative images comparing AngII treated aortae compared to age matched no angiotensin II control group. (B) Data for physical parameters of aortae, comparing AngII treated aortae from CCN4+/+ApoE-/- and CCN4-/-ApoE-/- mice with age matched no angiotensin II control group from CCN4+/+ApoE-/- and CCN4-/-ApoE-/- mice. Age matched controls receiving no angiotensin II showed no change in physical parameters between the CCN4+/+ApoE-/- and CCN4-/-ApoE-/- mice. (C) ApoE-/-CCN4-/- and ApoE-/-CCN4+/+ mice were exposed to AngII for 28 days. Apoptosis was quantified in aortae by immunohistochemistry for cleaved caspase 3. Positive cells are green and indicated with arrowheads (JPG 109 kb)

Dual staining showing co-localisation of apoptosis (cleaved PARP), proliferation (PCNA) and CCN4 with VSMCs (α-smooth muscle actin). Green indicates cleaved PARP, PCNA or CCN4 proteins, red indicates α-smooth muscle actin, and nuclei are stained blue with DAPI. IgG negative controls are included to demonstrate specificity of immunofluorescence protocol. Arrowheads indicate some positive cells (JPG 124 kb)

Immunofluorescence for CCN4 in aortae from CCN4+/+ApoE-/- and CCN4-/-ApoE-/- mice treated with AngII and CCN4+/+ApoE-/- mice without AngII. IgG negative controls are included to demonstrate specificity of immunofluorescence protocol. Green indicates CCN4 protein and nuclei are stained blue with DAPI. Arrowheads indicate some CCN4 positive cells (JPG 50 kb)

Western blotting for ICAM-1 and VCAM-1 proteins in endothelial cells cultured in the presence (CCN4) or absence (Con) of CCN4 protein for 24 hours. Molecular weights of detected proteins are indicated on right-handside of blot and stain-free band is shown as loading control (JPG 24 kb)

## Data Availability

Data is available on request.

## Abbreviations

CCN: CTGF, Cyr61, Nov
Cyr61: Cysteine rich angiogenic inducer 1
CTGF: Connective tissue growth factor
Nov: Nephroblastoma overexpressed gene
WISP-1: Wnt1 inducible signalling pathway protein 1
VSMCs: Vascular smooth muscle cells
TNFα: Tumour necrosis factor alpha
IL-10/18: Interleukin 10/18
TCF/LEF: T-cell factor/lymphoid enhancer factor
AP-1: Activator protein 1
MMP: Matrix metalloproteinase
CREB: CAMP response element binding protein
Syk: Spleen tyrosine kinase
PKCδ: Protein kinase C delta
JNK: C-Jun N terminal kinase
ICAM-1: ICAM-1
VCAM-1: Vascular cell adhesion protein 1
IL-6: Interleukin-6
HDACs: Histone deacetylases
AngII: Angiotensin II
EVG: Elastin Van Gieson
DAPI: 4′,6-Diaminidino-2-phenylindole
GSL: Griffonia simplicifolia lectin
PCNA: Proliferating cell nuclear antigen
PARP: Poly ADP ribose polymerase
IgG: Immunoglobulin G
M-CSF: Macrophage colony stimulating factor
FCS: Foetal calf serum
MCP-1: Monocyte chemoattractant protein 1
