# Supplementary figures and images for: Aneurysm severity is suppressed by deletion of CCN4
Source: J Cell Commun Signal. 2021 Jun 2;15(3):421–32. doi: 10.1007/s12079-021-00623-5 (PMC8222476; doi:10.1007/s12079-021-00623-5)

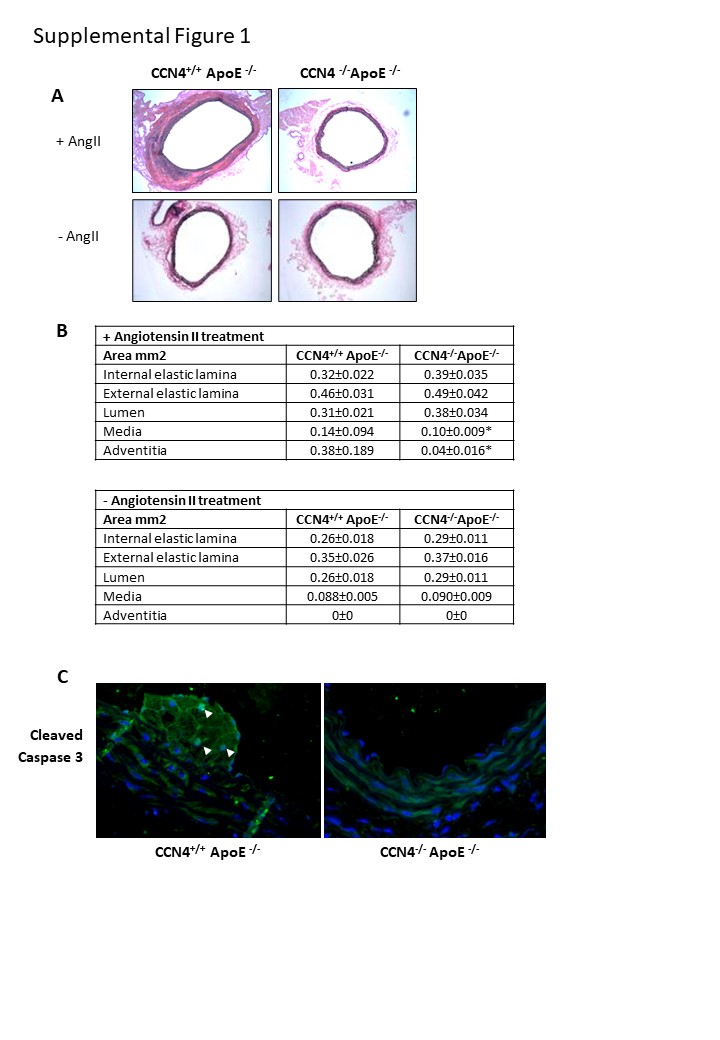

Supplement: Supplementary file 1 — (A) Representative images comparing AngII treated aortae compared to age matched no angiotensin II control group. (B) Data for physical parameters of aortae, comparing AngII treated aortae from CCN4+/+ApoE-/- and CCN4-/-ApoE-/- mice with age matched no angiotensin II control group from CCN4+/+ApoE-/- and CCN4-/-ApoE-/- mice. Age matched controls receiving no angiotensin II showed no change in physical parameters between the CCN4+/+ApoE-/- and CCN4-/-ApoE-/- mice. (C) ApoE-/-CCN4-/- and ApoE-/-CCN4+/+ mice were exposed to AngII for 28 days. Apoptosis was quantified in aortae by immunohistochemistry for cleaved caspase 3. Positive cells are green and indicated with arrowheads (JPG 109 kb) [file 12079_2021_623_MOESM1_ESM.jpg]

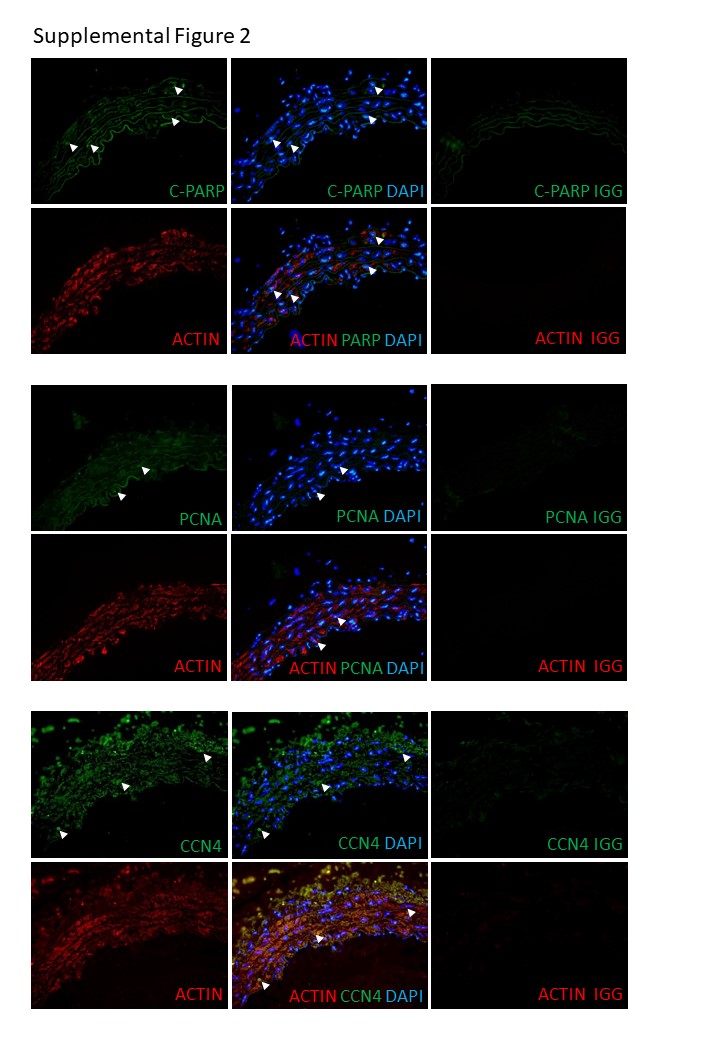

Supplement: Supplementary file 2 — Dual staining showing co-localisation of apoptosis (cleaved PARP), proliferation (PCNA) and CCN4 with VSMCs (α-smooth muscle actin). Green indicates cleaved PARP, PCNA or CCN4 proteins, red indicates α-smooth muscle actin, and nuclei are stained blue with DAPI. IgG negative controls are included to demonstrate specificity of immunofluorescence protocol. Arrowheads indicate some positive cells (JPG 124 kb) [file 12079_2021_623_MOESM2_ESM.jpg]

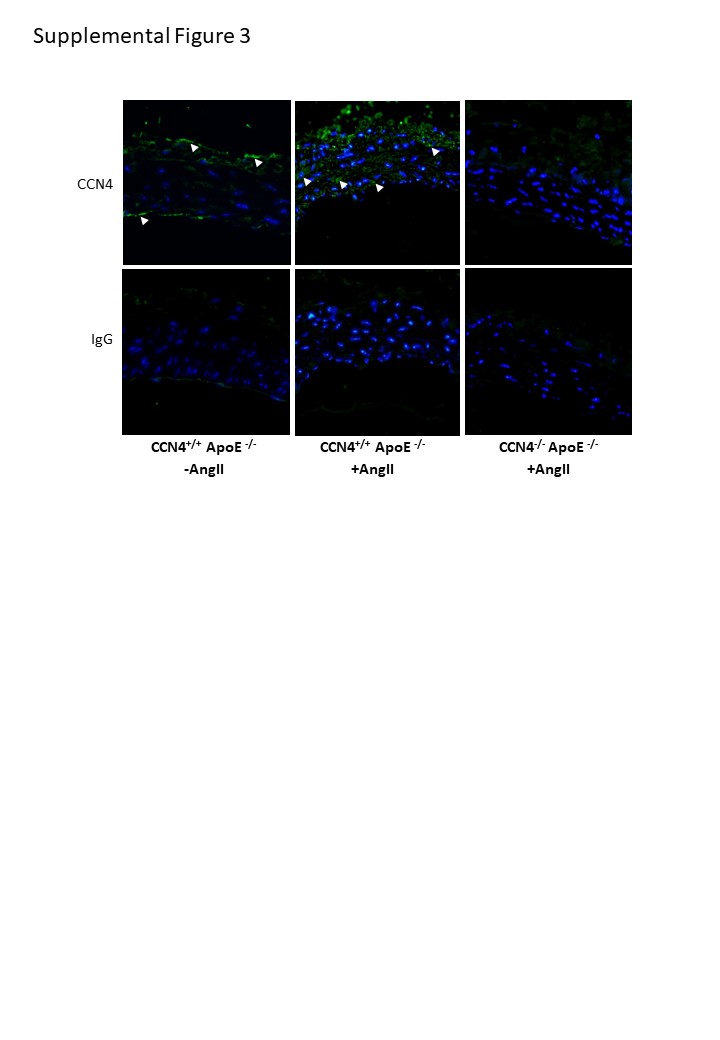

Supplement: Supplementary file 3 — Immunofluorescence for CCN4 in aortae from CCN4+/+ApoE-/- and CCN4-/-ApoE-/- mice treated with AngII and CCN4+/+ApoE-/- mice without AngII. IgG negative controls are included to demonstrate specificity of immunofluorescence protocol. Green indicates CCN4 protein and nuclei are stained blue with DAPI. Arrowheads indicate some CCN4 positive cells (JPG 50 kb) [file 12079_2021_623_MOESM3_ESM.jpg]

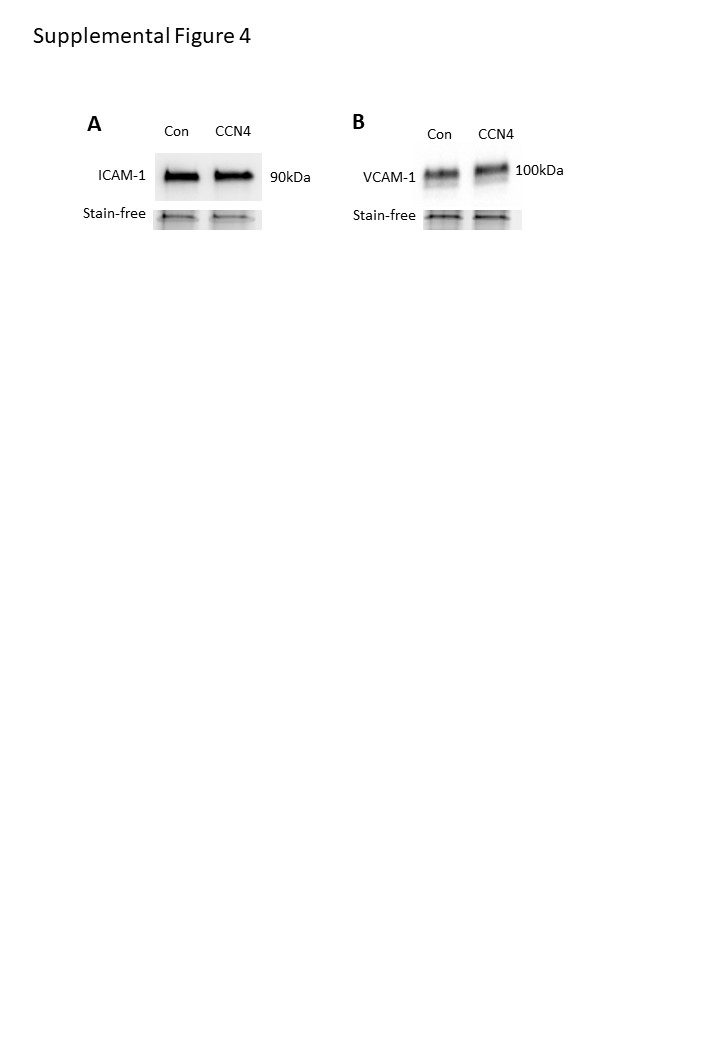

Supplement: Supplementary file 4 — Western blotting for ICAM-1 and VCAM-1 proteins in endothelial cells cultured in the presence (CCN4) or absence (Con) of CCN4 protein for 24 hours. Molecular weights of detected proteins are indicated on right-handside of blot and stain-free band is shown as loading control (JPG 24 kb) [file 12079_2021_623_MOESM4_ESM.jpg]
